# Stimulating EPA Biosynthesis in *Microchloropsis salina* through Cultivation with Selected Myxobacterial Culture Supernatants

**DOI:** 10.4014/jmb.2507.07041

**Published:** 2025-11-27

**Authors:** Seungjib Jeon, Buyng Su Hwang, Hyun Gi Koh, Bongsoo Lee

**Affiliations:** 1 Department of Chemical and Biomolecular Engineering, Korea Advanced Institute of Science and Technology (KAIST), Daejeon 34141, Republic of Korea; 2 Nakdonggang National Institute of Biological Resources (NNIBR), Sangju-si 37242, Republic of Korea; 3 Department of Biological and Chemical Engineering, College of Science and Technology, Hongik University, Sejong-si 30016, Republic of Korea; 4 Department of Microbial Biotechnology, College of Science and Technology, Mokwon University, Daejeon 35349, Republic of Korea

**Keywords:** *Microchloropsis salina*, EPA, omega-3, myxobacteria

## Abstract

Microalgae have emerged as a promising and sustainable platform for the production of omega-3 polyunsaturated fatty acids, offering an alternative to fish-derived sources. Among these, *Microchloropsis salina* (formerly *Nannochloropsis salina*) is particularly attractive as a candidate due to its naturally high eicosapentaenoic acid (EPA) content. In this study, bacterial supernatants from various myxobacterial strains were evaluated for their ability to enhance microalgal growth and EPA production. Remarkably, the supernatant derived from *Nannocystis* sp. KYC 2844 significantly improved biomass accumulation when added at 20% (v/v) to f/2 medium, increasing dry cell weight from 1.69 g/l (control) to 2.13 g/l. Although total lipid content decreased from 25.1% to 13.0%, the EPA fraction within the lipid markedly increased from 9.46% to 27.2%. As a result, the overall EPA titer reached 75.5 mg/l, representing a 1.87-fold improvement over the control. Subsequent nutrient analysis revealed that the KYC 2844 supernatant contained 127.3 ppm of ammonium (NH_4_^+^), which served as a preferred nitrogen source for *M. salina* and delayed nitrate utilization. These findings indicate that nitrogen speciation plays a critical role in shaping microalgal lipid profiles. Collectively, this study demonstrates that microbial supernatants can serve as effective medium supplements to enhance both growth and EPA productivity in microalgae and offers a potential strategy for improving the efficiency of microalgal-based omega-3 production systems.

## Introduction

Three main omega-3 unsaturated fatty acids, namely alpha-linolenic acid (ALA, C18:3), eicosapentaenoic acid (EPA, C20:5), and docosahexaenoic acid (DHA, C22:6), play essential roles in human nutrition [1, 2]. These fatty acids are involved in various biological processes, including the development of the nervous system, maintenance of vascular health, and regulation of immune and endocrine functions [3, 4]. However, as the human body cannot produce them on its own, these essential fatty acids must be obtained through dietary sources.

While ALA is abundant in plant-based sources such as nuts and seeds, EPA and DHA are found primarily in fish and other marine organisms [5]. With growing awareness of their health benefits, fish oil-based supplements are in increasing demand. However, overexploitation of fisheries has raised concerns about the long-term sustainability of this supply. Furthermore, in fact, the omega-3 fatty acids found in fish originate from microalgae, which form the base of the marine food web. This makes microalgae a sustainable and promising alternative for EPA and DHA production [6]. Species such as *Schizochytrium* and *Aurantiochytrium* are already used for DHA production [7-9], and microalgae can be cultivated using waste-derived nutrients, offering both economic and environmental advantages [10, 11].

One species of particular interest is *Microchloropsis* sp., which is among the most extensively studied marine microalgae. As an oleaginous species, it possesses high lipid content and productivity and is particularly known for its ability to accumulate elevated levels of EPA. Due to its potential in biodiesel production, genetic engineering efforts have been actively pursued, especially in *Microchloropsis salina* and *Nannochloropsis oceanica* [12-15]. The availability of genomic resources and molecular toolkits further supports its use as a platform for strain improvement [16, 17]. In addition, *Microchloropsis* is already widely cultivated in the aquaculture industry as a feedstock for farmed fish. These characteristics make it a highly attractive candidate for EPA production, either to enhance the omega-3 content of aquaculture products or for direct extraction as a dietary supplement.

Despite its potential as a platform for EPA production, *Microchloropsis* remains limited by high production costs, which hinder its competitiveness with fish oil in the commercial market [18]. To address this challenge, extensive research has focused on increasing lipid and polyunsaturated fatty acid productivity, often in the context of microalgal biofuel development. One widely studied strategy involves applying stress conditions, particularly nitrogen or phosphorus deprivation, to trigger lipid accumulation [17, 19, 20]. However, this typically results in reduced biomass productivity, limiting the overall improvement in lipid titer. To overcome this limitation, recent studies have increasingly applied multi-omics approaches, including lipidomics, metabolomics, and transcriptomics, to elucidate how cellular and molecular changes under various conditions influence lipid accumulation and EPA content, ultimately aiming to enhance overall productivity [17, 21-23].

In parallel, it has been reported that certain bacterial populations can stimulate microalgal growth through symbiotic interactions by supplying essential nutrients and producing growth-promoting factors such as phytohormones [24, 25]. Furthermore, other studies have shown that microalgae co-cultivated with bacteria exhibit enhanced growth compared to axenic cultures [26, 27]. In the open ocean, diverse organic materials secreted by animals, plants, bacteria, and viruses can aggregate with microalgae to form complex particulate structures [26]. In such environments, maintaining beneficial bacterial populations while suppressing harmful ones through competitive interactions is particularly important, especially since it is virtually impossible to sustain bacteria-free microalgal cultures in open pond systems [28]. These microbial dynamics should therefore be considered when developing strategies for microalgae-based lipid production.

Among various bacterial taxa, myxobacteria are notable for their ability to produce a wide range of secondary metabolites and large quantities of exopolysaccharides, which are known to influence the growth of other microorganisms either positively or negatively [18]. In the present study, the potential of these myxobacterial metabolites to enhance the biomass, lipid, and EPA productivity of *Microchloropsis salina* was investigated. Several myxobacterial strains were isolated and cultured, and their culture supernatants were tested for growth-promoting activity. In addition, nutrient stress experiments were conducted in combination with the addition of bioactive compounds to evaluate their synergistic effects on omega-3 lipid production in *M. salina*.

## Materials and Methods

### Microalgae Strain and Growth Conditions

*Microchloropsis salina* CCMP 1776, purchased from the National Center for Marine Algae and Microbiota (Bigelow Laboratory for Ocean Sciences, USA), was grown photoautotrophically in f/2 medium [29], containing the following component : NaNO_3_ (427.5 mg/l); NaH_2_PO_4_∙2H_2_O (30 mg/l); Tris-HCl (pH 7.6, 10 mM); 5 ml of trace metal composed of Na_2_EDTA∙2H_2_O (4.36 g/l), FeCl_3_∙6H_2_O (3.15 g/l), CoCl_2_∙ 6H_2_O (10 mg/l), ZnSO_4_∙7H_2_O (22 mg/l), MnCl_2_∙4H_2_O (180 mg/l), CuSO_4_∙5H_2_O (9.8 mg/l), Na_2_MoO_4_∙2H_2_O (6.3 mg/l) and 2.5 ml/l of vitamin stock composed of vitamin B_12_ (1 mg/l), Biotin (1 mg/l), thiamine∙HCl (200 mg/l) [30] in water including sea salt (15 g/l) (Sigma-Aldrich, USA). Cells were cultivated in 200 ml of f/2 medium at 25°C in 250 ml Erlenmeyer baffled flasks with shaking at 120 rpm under 120 μmol photons/m^2^/s of light intensity. The culture was mixed by aeration with a constant flow of 0.5 vvm (volume of air added to liquid volume per minute) air enriched with 2% CO_2_. Agar plates were prepared with 0.8% Bacto agar (Becton Dickinson, Republic of Korea) in an f/2 medium. For nitrogen limitation, f/2LN medium was prepared by reducing the NaNO_3_ concentration to 75 mg/l, while keeping all other components identical to the standard f/2 medium.

### Bacteria Strains and Growth Conditions

*Myxococcus fulvus* (KYC 1261), *M. virescens* (KYC 1262, 1264, 2003), *M. stipitatus* (KYC2118, 1127, 1001) *Cystobacter* (KYC 1668), and *Nannocystis* (KYC 2818, 2844) obtained from Myxobank (Republic of Korea) were used in this study. Cells were cultivated in Casitone–Yeast Extract (CYE) medium [31] containing the following components: MgSO_4_∙7H_2_O (1 g/l); Bacto Casitone (10 g/l); Bacto Yeast Extract (5 g/l), and Vitamin B_12_ (5 mg/l) in distilled water. To maintain pH 7.6, cultures were diluted in 10 mM morpholinepropanesulfonic acid (MOPS) buffer. To obtain colonies from bacteria stock, CYE agars were prepared with 1.5% Bacto agar. The cells were grown at 32°C with shaking at 160 rpm without fluorescent light.

### Cultivation of *Microchloropsis salina* with Bacterial Supernatant

To obtain the bacterial supernatant, bacteria were grown in CYE broth medium at 32°C with shaking at 160 rpm for two days and the culture was centrifuged at 7,000 rpm for 5 min at 4°C. The resulting supernatant was filtered through a 0.22 μm syringe filter (Sartorius Stedim, USA). Conditioned media containing bacterial supernatant consisted of 80–95% f/2 medium and 5–20% bacterial supernatant. The *M. salina* cells were inoculated at an optical density (OD_680_) of 0.2 and cells were cultivated in 200 ml conditioned medium at 25°C with shaking at 120 rpm under continuous illumination at 120 μmol photons/m^2^/s. All experiments were conducted in three independent biological replicates, except for the preliminary screening experiments involving multiple myxobacterial strains.

### Nutrient Analysis of Broth Culture

To identify the consumption of nutrients in the culture, 2 ml cultures were collected in 15 ml Falcon tubes. The samples were filtered through a 0.22 μm syringe filter (Sartorius Stedim) and then stored at -4°C. Analysis of nitrate (NO_3_^-^), phosphate (PO_4_^3-^), and ammonium (NH_4_^+^) was conducted using ion chromatography (881 compact IC pro, Metrohm, Switzerland) with a Metrosep A Supp5 150 column for anions. The amino acid composition was analyzed by an amino acid analyzer (L-8900, Hitachi, Japan) based on a post-column derivatization method with ninhydrin, and a total of 17 amino acids were quantified, including aspartic acid (Asp), threonine (Thr), serine (Ser), glutamic acid (Glu), glycine (Gly), alanine (Ala), cysteine (Cys), valine (Val), methionine (Met), isoleucine (Ile), leucine (Leu), tyrosine (Tyr), phenylalanine (Phe), lysine (Lys), histidine (His), tryptophan (Trp), and arginine (Arg). The concentrations of amino acids were determined using an external standard calibration method.

### Growth and Cell Size Analysis

Cell growth of *M. salina* during cultivation was assessed by optical density, dry cell weight, and cell counting. For optical density measurement, 1 ml of culture was measured at 680 nm after appropriate dilution using a UV spectrophotometer (Beckman-Coulter, USA). For dry cell weight, 5 ml of culture was centrifuged at 13,000 rpm for 5 min at 25°C in microtubes (Axygen, USA) after being washed with distilled water. The pellets were dried in an oven at 105°C for 24 h, and the dry biomass was weighed.

Cell counting and size measurements were performed by pipetting a 20 μl of each culture onto a Cellometer Auto X4 Cell Counter (Nexcelom Bioscience, USA). Additionally, cell size was cross validated using a flow cytometry with a MoFlo XDP system (Beckman Coulter, USA), which is equipped with a 488 nm blue laser for forward and side scatter detection.

### Fatty Acid Methyl Ester (FAME) Analysis

Cells were harvested by centrifugation at 4,000 rpm for 15 min (Supera-22K, Hanil Science Industrial, Republic of Korea), and then washed with distilled water to remove sea salts. The harvested cells were freeze-dried at −40°C for 72 h (FD5508, IlShin BioBase, Republic of Korea). To extract lipids from the lyophilized biomass, a chloroform–methanol mixture (2:1, v/v) was used following a modified Folch method [32]. The extracted lipids were converted to FAMEs by methanolysis with methanol and sulfuric acid at 100°C for 20 min. After the reaction, distilled water was added to separate the phases, and the samples were centrifuged at 4,000 rpm for 10 min at 20°C to obtain an organic phase containing FAMEs. The content and composition of FAMEs were analyzed by gas chromatography (HP 6890, Agilent, USA) using a flame ionization detector (FID).

## Results and Discussion

### Screening of Different myx

**Obacterial strains.** To identify myxobacterial strains whose culture supernatants have meaningful effects on the cultivation of *M. salina*, six strains were tested: *Myxococcus stipitatus* (KYC 1001), *Myxococcus fulvus* (KYC 1264), *Myxococcus xanthus* (KYC 2065), *Cyanobacterium* sp. (KYC 1668), *Nannocystis* sp. (KYC 2844), and *Polyangium* sp. (KYC 2818). To assess the effects of bacterial metabolites, 10% (v/v) culture supernatants from each strain grown in CYE medium were added to the f/2 medium for *M. salina* cultivation. CYE medium and deionized water were used as controls to exclude effects unrelated to bacterial metabolites.

**Fig. 1:**
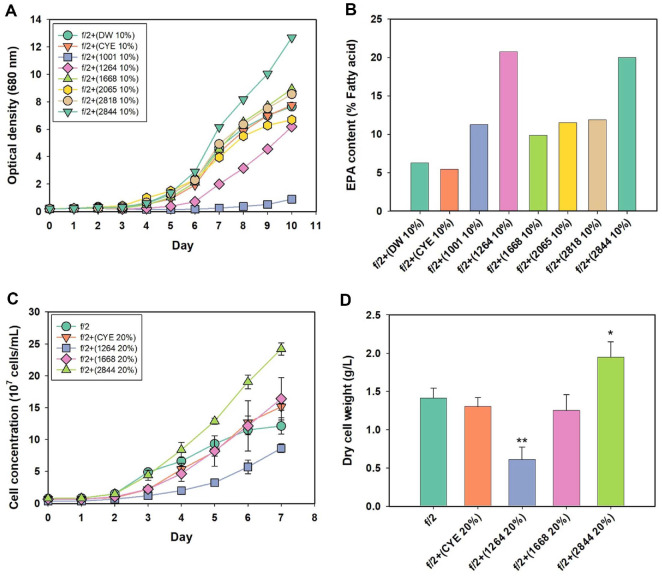
Effects of different myxobacterial supernatants on the growth and EPA content of *M. salina*. (**A**) Growth curves of *M. salina* cultivated for 10 days in f/2 medium supplemented with 10% DW, CYE or bacterial supernatants. (**B**) EPA content expressed as a percentage of total fatty acids in *M. salina* harvested under the same conditions. (**C**) Growth curves of *M. salina* cultivated for 7 days in f/2 medium supplemented with 20% culture supernatants from selected strains (KYC 1264, KYC 1668, and KYC 2844) or 20% CYE medium. (**D**) Dry cell weight (DCW) of *M. salina* after 7 days of cultivation under the same conditions. Data for (**C**) and (**D**) represent the mean ± standard deviation (*n* = 3). Statistical significance was assessed using Student’s *t*-test, and significant differences were indicated by asterisks (*p < 0.05, **p < 0.01, ***p < 0.001)

As shown in Fig. 1A, the addition of 10 % CYE medium to f/2 did not result in any significant difference in algal growth compared to the DW control, indicating that the CYE medium itself had no notable effect on *M. salina*. In contrast, supplementation with myxobacterial supernatants led to distinct differences in growth. Strains KYC 2844, KYC 1668, and KYC 2818 promoted growth, whereas KYC 2065, KYC 1264, and KYC 1001 suppressed it (Fig. 1A). Notably, the supernatant from *Nannocystis* sp. (KYC 2844) resulted in a pronounced increase in growth, with OD_600_ reaching 12.7 compared to 7.65 in the control. This was accompanied by a higher biomass titer, reaching 2.98 g/l, representing a 20 % increase over the control (2.48 g/l) (Table S1). Cultures treated with KYC 1668 and KYC 2818 supernatants also showed elevated optical densities, reaching 8.92 and 8.56, respectively. However, the final biomass titer for these conditions were 2.50 g/l and 2.24 g/l, which were comparable to the control and did not reflect a substantial increase.

In contrast, algal growth was severely inhibited in cultures supplemented with the supernatant from *Myxococcus stipitatus* (KYC 1001). The final optical density reached only 0.900, which was markedly lower than in all other conditions, indicating a strong suppressive effect on cell proliferation. This inhibitory effect is likely attributed to secondary metabolites secreted by KYC 1001 that exert algicidal or cytotoxic activity. It is well documented that certain myxobacteria produce diverse bioactive compounds capable of lysing not only other bacteria but also microalgae [33]. Given that various chemical compounds have been reported to regulate microalgal growth and lipid accumulation [34, 35], metabolites from KYC 1001 may similarly influence algal physiology, either by suppressing growth or by promoting lipid accumulation during the stationary phase.

Beyond growth effects, the supernatants were also evaluated for their impact on EPA accumulation in algal lipids (Fig. 1B). As expected, cultures treated with CYE, which resulted in an EPA content of 5.44% of total fatty acids, showed no significant difference compared to those treated with DW, which had an EPA content of 6.25%. This confirms that CYE itself had no direct effect on EPA biosynthesis. In contrast, the addition of myxobacterial supernatants led to increased EPA content in all tested conditions. In particular, KYC 1264 and KYC 2844 induced substantial EPA accumulation, reaching 20.75% and 19.98% of total fatty acids, respectively, representing more than a threefold increase compared to the control. Given that the fatty acid profile in *Microchloropsis* species is influenced by environmental stresses such as light, pH, and temperature [23], it is likely that the bacterial supernatants imposed a stress condition favorable for EPA biosynthesis. This approach provides a promising strategy for enhancing EPA production without relying on nutrient deprivation or genetic modification.

Based on the results of growth and lipid profiling, KYC 1264, KYC 1668, and KYC 2844 were selected for further evaluation due to their positive effects on either algal growth or EPA accumulation. To better elucidate the impact of each supernatant, the concentration was elevated to 20% (v/v), and the cultures were re-evaluated under the same conditions as before (Fig. 1C and 1D). Among the three, the culture supplemented with the supernatant from KYC 2844 produced the highest biomass, reaching 1.95 g/l, which corresponds to a 38% increase compared to the control (1.41 g/l). In contrast, cultures treated with KYC 1264 and KYC 1668 at the same concentration exhibited growth inhibition, yielding only 0.61 g/l and 1.25 g/l of biomass, respectively. Based on these findings, KYC 2844 was ultimately selected as the most promising strain for subsequent experiments, as it demonstrated beneficial effects on both biomass accumulation and EPA enrichment.

### Effects of Bacterial Metabolites and Nitrogen Availability on *Microchloropsis salina*

**Fig. 2:**
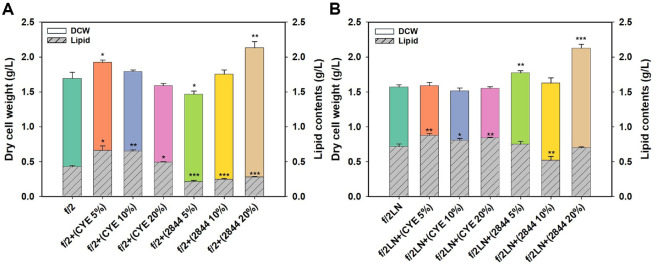
Effects of varying concentrations of CYE medium and KYC 2844 supernatant on biomass and lipid production in *M. salina*. (**A**) Dry cell weight (DCW, solid bars) and lipid content (hatched bars) of *M. salina* after 7 days of cultivation in standard f/2 medium supplemented with 5%, 10%, or 20% (v/v) of either CYE medium or KYC 2844 supernatant. (**B**) DCW and lipid content of *M. salina* under nitrogen-limited conditions (f/2LN), cultivated with the same supplementation levels of CYE medium or KYC 2844 supernatant as in (**A**). In both panels, lipid content is represented as the shaded portion of the total DCW. Data are shown as mean ± standard deviation from biological triplicates (*n* = 3). Statistical significance was assessed using Student’s *t*-test, and significant differences were indicated by asterisks (*p < 0.05, **p < 0.01, ***p < 0.001)

In microalgae, lipid biosynthesis is typically activated under stress conditions, often resulting in an inverse relationship between biomass productivity and lipid accumulation. Following the preliminary screening, *Nannocystis* sp. KYC 2844 was identified as the most promising strain for enhancing EPA production in *M. salina*. To investigate the concentration-dependent effects of its metabolites, the culture supernatant was tested at 5%, 10%, and 20% (v/v). Given the well-established influence of nitrogen availability on both algal growth and lipid biosynthesis, each condition was evaluated under nitrogen-sufficient (f/2) and nitrogen-limited (f/2LN) media (Fig. 2).

On day 7 of cultivation under nitrogen-sufficient conditions (f/2), dry cell weight (DCW) responded differently to supplementation with CYE medium and the KYC 2844 culture supernatant (Fig. 2A). Notably, while low concentrations of CYE (5% and 10%) enhanced DCW, this positive effect progressively diminished as the concentration increased. In contrast, supplementation with the KYC 2844 culture supernatant led to a concentration-dependent increase in DCW. The highest DCW was observed in cultures supplemented with 20%supernatant, reaching 2.13 g/l, which corresponds to a 25.8% increase compared to the f/2 control (1.69 g/l). These results further support the presence of growth-promoting metabolites in the *Nannocystis* sp. KYC 2844 supernatant (Fig. 1). In contrast, the mechanism underlying the growth-promoting effect of CYE remains unclear. It is presumed that the complex composition containing casitone and yeast extract provides additional nutrients such as amino acids and trace elements that support algal growth at low concentrations [36, 37]. However, as the proportion of CYE increases, the relative amount of f/2 medium decreases, potentially leading to salinity or nutrient imbalances that counteract the initial growth-promoting effects.

Under nitrogen-limited conditions (f/2LN), cultures without any supplementation exhibited an 7.1% lower dry cell weight (DCW) compared to those grown in nitrogen-sufficient medium (f/2), reflecting typical growth suppression associated with nitrogen deficiency (Fig. 2B). However, the relatively small difference suggests that nitrogen may have been depleted during the f/2 condition as well. Notably, when 20% CYE or 20% *Nannocystis* sp. KYC 2844 culture supernatant was added, the DCW values under both f/2 and f/2LN conditions were nearly identical. In the case of CYE, this effect may be attributed to nitrogenous compounds such as amino acids derived from casitone and yeast extract, which could have partially compensated for nitrogen limitation [37]. Furthermore, since high concentrations of CYE tended to reduce DCW even under nitrogen-sufficient conditions, the negative effects of CYE supplementation may have masked any differences due to nitrogen availability. In contrast, the comparable DCW observed in cultures supplemented with 20% KYC 2844 supernatant under both f/2 and f/2LN conditions implies that the supernatant contains bioactive metabolites that can mitigate nitrogen stress or provide alternative nitrogen sources.

In addition to cell growth, nitrogen availability and supplementation also had a pronounced impact on lipid accumulation in *M. salina*. As expected, cultures grown under nitrogen-limited conditions (f/2LN) exhibited significantly higher lipid content across all tested conditions compared to their nitrogen-sufficient (f/2) counterparts (Fig. 2A and 2B, Table S2) [38, 39]. Supplementation with either CYE or the KYC 2844 supernatant markedly influenced the proportion of lipids relative to biomass. In f/2 medium without supplementation, lipids accounted for approximately 25.1% of the dry cell weight. However, the addition of 5–20% CYE increased the lipid content to 31–36%, suggesting that complex nutrients in CYE may stimulate lipid biosynthesis. In contrast, cultures supplemented with the KYC 2844 supernatant showed a significant reduction in lipid content, with values ranging from 13% to 14%, implying a potential trade-off between enhanced growth and lipid accumulation. A similar trend was observed under nitrogen-limited conditions (Fig. 2B). In f/2LN without supplementation, the lipid content reached 45.77%, and increased slightly to 53–55% with the addition of CYE, peaking at 55.34% with 5% CYE. In contrast, supplementation with the KYC 2844 supernatant led to a concentration-dependent reduction in lipid accumulation. The addition of 5% supernatant resulted in a moderate decrease to 42.41%, while 10% and 20% supplementation further reduced the lipid content to approximately 32%. These results indicate that although the KYC 2844 supernatant enhances biomass production, it may concurrently suppress lipid biosynthesis, particularly under nitrogen-limited conditions.

Along with changes in total lipid content, the fatty acid composition of the accumulated lipids also varied significantly depending on the supplement used (Table 1 and Fig. S1). In particular, the proportion of eicosapentaenoic acid (EPA) showed the most notable variation. Under nitrogen-sufficient (f/2) conditions, EPA accounted for 9.46% of the total lipid content in the control group. This proportion decreased upon CYE supplementation, reaching a minimum of 6.38% with 5% CYE. In contrast, cultures supplemented with the KYC 2844 supernatant exhibited a concentration-dependent increase in EPA content, with the highest value observed at 27.2% under 20% supplementation. This corresponds to nearly a 2.88-fold increase compared to the control. Although the overall lipid content in KYC 2844-supplemented cultures was relatively low, the marked increase in EPA proportion led to a significantly higher total EPA titer. In particular, cultures grown in f/2 medium with 20% KYC 2844 supernatant achieved an EPA titer of 75.5 mg/l, which is 1.87 times higher than that of the f/2 control. Under nitrogen-limited (f/2LN) conditions, the EPA proportion remained low across all conditions, ranging from 2% to 6%. Nonetheless, the addition of 20% KYC 2844 supernatant resulted in the highest EPA content in this group (6.01%). This decrease is likely attributable to the fact that EPA in *M. salina* predominantly exists in the form of glycolipids and phospholipids, both of which require nitrogen and phosphorus for synthesis [17, 23]. Despite the elevated total lipid accumulation typically observed under nitrogen-limited conditions, the reduced EPA proportion ultimately led to lower overall EPA titer compared to nitrogen-sufficient conditions [40]. These findings indicate that while nitrogen limitation enhances total lipid content, adequate nitrogen availability is essential for maximizing EPA productivity.

### Nutrient Contents of Cultures and Nitrogen Source Preference by Microalgae

**Fig. 3:**
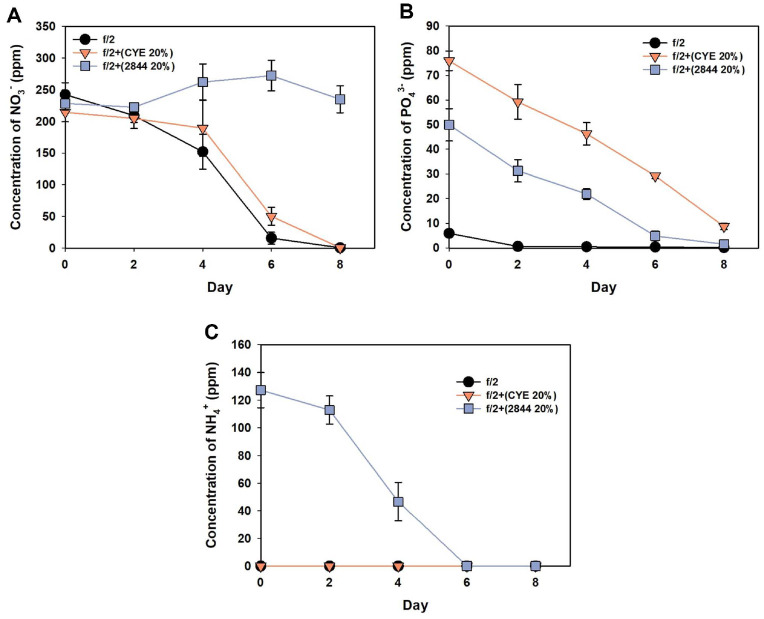
Nitrogen and phosphate dynamics in cultures of *M. salina* supplemented with CYE medium or KYC 2844 supernatant. (**A**) Nitrate (NO_3_^-^), (**B**) phosphate (PO_4_^3-^), and (**C**) ammonium (NH_4_^+^) concentrations were monitored over 8 days in f/2 medium, f/2 supplemented with 20% CYE medium, and f/2 supplemented with 20% KYC 2844 supernatant. Error bars indicate mean ± standard deviation from biological triplicates (*n* = 3).

To explore the underlying reason behind the distinctive effects of KYC 2844 supernatant supplementation on EPA production, nutrient levels in the culture media were analyzed by ion chromatography, with a particular focus on nitrogen and phosphorus species (Fig. 3). Since the condition with 20% KYC 2844 supernatant yielded the highest EPA productivity in earlier experiments, nutrient analysis was conducted specifically for f/2 medium, f/2 with 20% CYE, and f/2 with 20% KYC 2844 supernatant.

In terms of nitrate (NO_3_^-^) dynamics, *M. salina* cultured in f/2 control medium immediately began assimilating nitrate from day 0, with levels gradually declining and becoming fully depleted by day 6 (Fig. 3A). In contrast, in the culture supplemented with 20% KYC 2844 supernatant, nitrate levels did not decline until day 6 and showed minimal utilization even by day 8. This observation supports the similar biomass outcomes under both f/2 and f/ 2LN conditions with KYC 2844 supernatant supplementation and strongly suggests the presence of additional nitrogen sources in the culture. Subsequent analysis confirmed the presence of 127.3 ppm ammonium (NH_4_^+^) in the culture supplemented with 20% KYC 2844 supernatant at day 0 (Fig. 3C). The ammonium concentration remained relatively constant until day 2, then began to decline rapidly and became undetectable by day 6. This temporal pattern aligns with the delayed onset of nitrate utilization, indicating that *M. salina* prioritized ammonium assimilation when both nitrogen sources were available. In the culture supplemented with 20% CYE, ammonium was not detected throughout the cultivation (Fig. 3B). In this condition, nitrate consumption began after day 4, implying that *M. salina* initially relied on more readily assimilable nitrogen sources such as amino acids, which may have originated from the CYE medium, before transitioning to nitrate uptake.

**Fig. 4:**
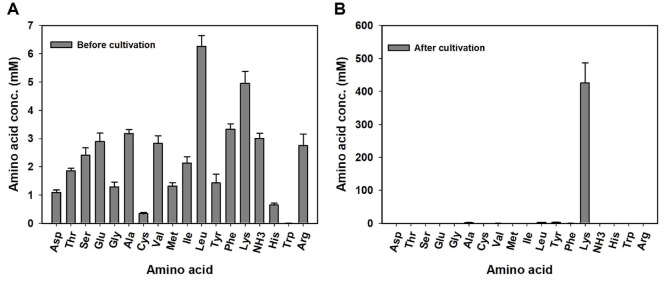
Amino acid depletion and ammonium accumulation in CYE medium before and after cultivation of *Nannocystis* sp. KYC 2844. (**A**) Chromatographic profile of fresh CYE medium showing a diverse array of amino acids and a low baseline level of ammonium. (**B**) After cultivation of *Nannocystis* sp. KYC 2844, most amino acids were depleted, and a sharp increase in the ammonium peak was observed. Error bars indicate mean ± standard deviation from biological triplicates (*n* = 3).

As neither the f/2 medium nor the CYE medium contains a significant amount of ammonium, the presence of ammonium in the KYC 2844-supplemented culture is likely attributable to the metabolic activity of the myxobacteria. It is plausible that the bacteria consumed amino acids during growth and released ammonium as a byproduct, which subsequently accumulated in the supernatant. To investigate this possibility, the composition of the KYC 2844 culture medium was examined both before and after bacterial growth. Analysis of the fresh CYE medium showed a diverse array of amino acids along with a low background level of ammonium (Figs. 4A and S2A). After cultivation with KYC 2844, however, most amino acids had been depleted, and a pronounced increase in ammonium concentration was observed (Figs. 4B and S2B). These findings suggest that the myxobacteria actively consumed amino acids during growth and released ammonium as a metabolic byproduct. As a result, the supernatant became enriched with ammonium, a nitrogen source that is readily assimilated by microalgae such as *Nannochloropsis* sp. [41, 42].

In contrast to the comparable nitrate concentrations initially observed across f/2, f/2 with 20% CYE, and f/2 with 20% KYC 2844 supernatant, phosphate levels varied substantially among the conditions. Due to the high phosphate content of the CYE medium [43], the culture supplemented with 20% CYE exhibited an initial phosphate concentration of 75.88 ppm, which was nearly 13 times greater than that of the f/2 control (5.91 ppm). As a result, significant amounts of phosphate remained unassimilated at the end of the cultivation period (Fig. 3B). Although the KYC 2844 supernatant had been partially depleted of phosphate through bacterial metabolism, the initial phosphate concentration in the 20% KYC 2844 condition was still high at 49.96 ppm.

The presence of both readily assimilable ammonium and abundant phosphate in the KYC 2844 supernatant-supplemented cultures provided a nutrient-rich environment that supported robust biomass accumulation. However, excessive nitrogen availability has been shown to suppress lipid accumulation in microalgae, which may explain the lower lipid content observed under this condition. Interestingly, despite the reduction in overall lipid content, the fraction of EPA within the lipids was significantly elevated. This suggests that nitrogen abundance may have contributed to a shift in lipid composition favoring EPA production [23].

Additional experiments using supernatants from KYC 1264 and KYC 1668 revealed that these myxobacterial strains also released substantial amounts of ammonium after depleting amino acids (Fig. S3). However, only KYC 2844 supernatant consistently led to both increased biomass and elevated EPA content. This indicates that other strain-specific metabolites, such as exopolysaccharides, may also contribute to the observed effects. Although the precise identity and mechanism of action of these bacterial metabolites remain to be elucidated, these findings highlight the potential of tailoring nitrogen source composition and leveraging beneficial microbial byproducts to enhance EPA production in *M. salina*.

These results collectively suggest that specific myxobacterial metabolites, particularly ammonium, can not only alleviate nitrogen limitation but also influence the lipid metabolic pathways of *M. salina* in a manner that favors EPA enrichment. By selectively supplementing cultures with well-characterized bacterial supernatants, it may be possible to decouple biomass growth from lipid repression and steer microalgal metabolism toward the production of EPA-rich lipids. This study underscores the feasibility of microbial co-culture or metabolite-guided supplementation strategies as a practical approach to improving the titer and quality of high-value products such as EPA in industrial microalgal cultivation.

## Conclusion

This study demonstrated that culture supernatants from myxobacteria can substantially influence both the growth and lipid profile of *M. salina*. Among the strains tested, the supernatant derived from *Nannocystis* sp. KYC 2844 markedly enhanced biomass accumulation and significantly increased the proportion of EPA within the lipid fraction. Notably, the EPA content in total lipids increased from 9.93% (control) to 26.37% in the presence of 20% KYC 2844 supernatant. Despite a lower total lipid content, this increase in EPA proportion led to an overall EPA titer of 75.5 mg/l, representing a 1.87-fold improvement compared to the control. Ion chromatography revealed that this effect was largely attributable to the presence of 127.3 ppm of ammonium (NH_4_^+^) in the supernatant, which served as a readily assimilable nitrogen source and delayed nitrate uptake during cultivation. These findings highlight the pivotal role of nitrogen speciation in shaping microalgal lipid composition and underscore the potential of microbial metabolites as effective medium supplements. Although the precise identity of other contributing bioactive compounds remains to be clarified, this study provides a foundation for optimizing microalgal EPA production through medium engineering and microbial co-culture strategies.

## Supplemental Materials

Supplementary data for this paper are available on-line only at http://jmb.or.kr.



## Figures and Tables

**Table 1:** EPA content and titer of *M. salina* cultivated for 7 days in f/2 and f/2LN (low nitrogen) media supplemented with conditioned supernatants.

Supplementation	f/2 medium		f/2LN medium	
	EPA content (%)	EPA titer (mg/l)	EPA content (%)	EPA titer (mg/l)
None	9.46 ± 1.09	40.3 ± 6.2	3.42 ± 0.21	24.6 ± 2.3
+CYE 5%	6.38 ± 0.15*	42.2 ± 4.9	3.21 ± 0.25	28.1 ± 1.7
+CYE 10%	7.42 ± 0.54*	48.2 ± 2.2	2.37 ± 0.10***	19.1 ± 0.2
+CYE 20%	8.13 ± 0.26	40.0 ± 1.4	3.45 ± 0.46	29.0 ± 3.6
+2844 5%	19.1 ± 0.3***	40.7 ± 3.7	3.86 ± 0.12*	29.1 ± 2.2
+2844 10%	22.2 ± 0.8***	54.6 ± 5.0*	5.42 ± 0.32**	28.2 ± 1.2
+2844 20%	27.2 ± 0.8***	75.5 ± 4.3***	6.01 ± 0.31***	42.0 ± 2.9**

Statistical significance was determined using Student’s *t*-test and is indicated by asterisks (*p < 0.05, **p < 0.01, and ***p < 0.001).
