# Supplementary material for: Stimulating EPA Biosynthesis in Microchloropsis salina through Cultivation with Selected Myxobacterial Culture Supernatants
Source: J Microbiol Biotechnol. 2025 Nov 27;35:e2507041. doi: 10.4014/jmb.2507.07041 (PMC12685597; doi:10.4014/jmb.2507.07041)
Supplement: Supplementary file 1 [file jmb-35-e2507041-supple.pdf]

J. Microbiol. Biotechnol. 2025. 35: e2507041

https://doi.org/10.4014/jmb.2507.07041

Supplementary Figures and Tables

Stimulating EPA Biosynthesis in *Microchloropsis salina* through Cultivation with Selected Myxobacterial Culture Supernatants

Seungjib Jeon[1†], Buyng Su Hwang[2†], Hyun Gi Koh[3*], and Bongsoo Lee[4*]

[1] Department of Chemical and Biomolecular Engineering, Korea Advanced Institute of Science and Technology (KAIST), 291 Daehak-ro, Yuseong-gu, Daejeon 34141, Republic of Korea

[2]Nakdonggang National Institute of Biological Resources (NNIBR), 137 Donam 2-gil, Sangju-si, Gyeongsangbuk-do, 37242, Republic of Korea

[3]Department of Biological and Chemical Engineering, College of Science and Technology, Hongik University, 2639 Sejong-ro, Sejong-si, 30016, Republic of Korea

[4]Department of Microbial Biotechnology, College of Science and Technology, Mokwon University, 88 Doanbuk-ro, Seo-Gu, Daejeon 35349, Republic of Korea

[†]Seungjib Jeon and Buyng Su Hwang contributed equally to this work.

***Correspondence:**

hgkoh@hongik.ac.kr, bongsoolee@mokwon.ac.kr

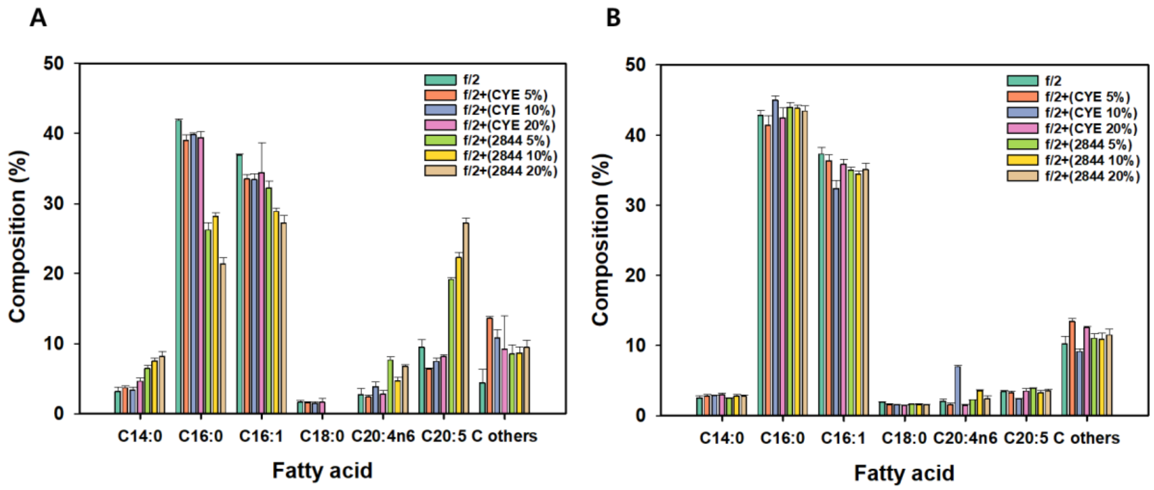

Fig. S1 Effects of CYE and 2844 supernatant supplementation on fatty acid composition. (A) Fatty acid profiles of microalgal cells cultivated in f/2 medium with or without supplementation of CYE or 2844 culture supernatant at 5%, 10%, and 20% concentrations. **(B)** Fatty acid profiles of total lipids extracted from microalgae grown under the same supplementation conditions in f/2LN medium. Error bars indicate mean ± standard deviation from biological triplicates (n = 3).

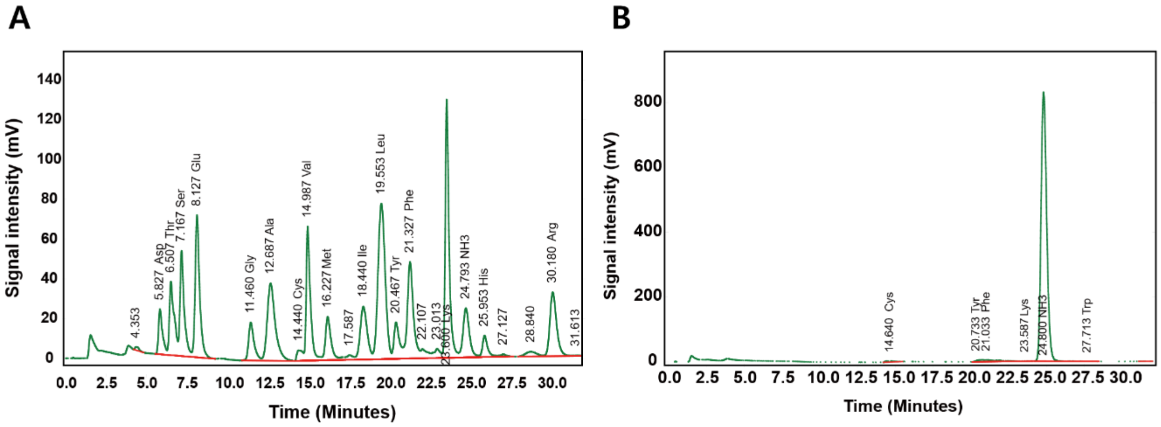

Fig. S2. Nitrogen and phosphate dynamics in cultures of *M. salina* supplemented with CYE medium or KYC 2844 supernatant. (A) Nitrate (NO_3^-), **(B)** phosphate (PO_4^{3-}), and **(C)** ammonium (NH_4^+) concentrations were monitored over 8 days in f/2 medium, f/2 supplemented with 20% CYE medium, and f/2 supplemented with 20% KYC 2844 supernatant.

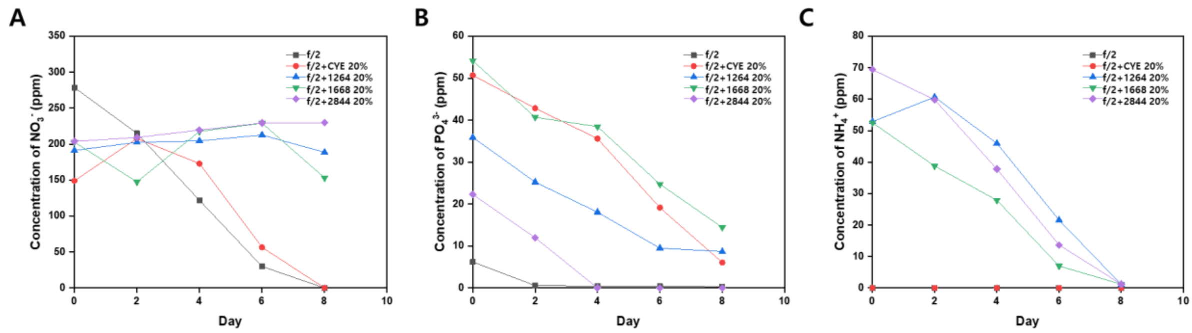

Fig. S3 Nitrogen and phosphate dynamics in cultures of *M. salina* supplemented with bacterial culture supernatants. **(A)** Nitrate (NO_3^-), **(B)** phosphate (PO_4^{3-}), and **(C)** ammonium (NH_4^+) concentrations were monitored over 8 days in f/2 medium, f/2 supplemented with 20% CYE medium, and f/2 supplemented with 20% culture supernatants from KYC 1264, KYC 1664, or KYC 2844.

Table S1. Dry cell weight of *M. salina* after 10 days of cultivation in f/2 medium supplemented with different concentrations of various bacterial supernatants during the initial screening experiment.

Day 10	f/2 + (DW 10%)	f/2 + (CYE 10%)	f/2 + (1001 10%)	f/2 + (1264 10%)	f/2 + (1668 10%)	f/2 + (2065 10%)	f/2 + (2818 10%)	f/2 + (2844 10%)
DCW (g/L)	2.48	2.48	0.32	1.88	2.50	1.86	2.24	2.98

Table S2. Dry cell weight and lipid content of *M. salina* cultivated for 7 days in f/2 medium supplemented with varying concentrations of CYE or KYC 2844 supernatant (n = 3). Statistical differences were assessed by Student's *t*-test, and significance levels were denoted by asterisks ($^{*}p < 0.05$, $^{**}p < 0.01$, and $^{***}p < 0.001$).

Supplementation	f/2 medium		f/2LN medium	
	DCW (g/L)	Lipid content (%)	DCW (g/L)	Lipid content (%)
none	1.69 ± 0.09	25.1 ± 1.6	1.57 ± 0.03	45.8 ± 1.2
+CYE 5%	1.92 ± 0.03*	34.3 ± 2.9*	1.59 ± 0.05	55.4 ± 2.5**
+CYE 10%	1.79 ± 0.02	36.3 ± 0.7***	1.51 ± 0.04	53.3 ± 1.5**
+CYE 20%	1.59 ± 0.03	31.0 ± 0.4**	1.55 ± 0.02	54.2 ± 1.1***
+2844 5%	1.47 ± 0.05*	14.5 ± 0.8***	1.77 ± 0.03**	42.4 ± 2.1
+2844 10%	1.75 ± 0.06	14.0 ± 0.4***	1.63 ± 0.07	32.1 ± 3.5**
+2844 20%	2.13 ± 0.09**	13.0 ± 0.1**	2.13 ± 0.06***	32.8 ± 0.4***
